# Insulin-like growth factor 1 and sex hormones for assessment of anthropometric and pubertal growth of Egyptian children and adolescents with type 1 diabetes mellitus (single center study)

**DOI:** 10.1186/s12902-024-01596-3

**Published:** 2024-05-09

**Authors:** Rasha A. Thabet, Eman M. Sherif, Ahmed O Abd ElAal, Rana A. Mahmoud

**Affiliations:** 1 Pediatrics, Faculty of Medicine, Ain Shams University, Cairo, Egypt; 2 Egypt Ministry of Health and Population, Cairo, Egypt

**Keywords:** Anthropometric measurements, Pubertal growth, T1DM

## Abstract

**Background:**

This study aimed to assess the anthropometric measures and pubertal growth of children and adolescents with Type 1 diabetes mellitus (T1DM) and to detect risk determinants affecting these measures and their link to glycemic control.

**Patients and methods:**

Two hundred children and adolescents were assessed using anthropometric measurements. Those with short stature were further evaluated using insulin-like growth factor 1 (IGF-1), bone age, and thyroid profile, while those with delayed puberty were evaluated using sex hormones and pituitary gonadotropins assay.

**Results:**

We found that 12.5% of our patients were short (height SDS < -2) and IGF-1 was less than -2 SD in 72% of them. Patients with short stature had earlier age of onset of diabetes, longer duration of diabetes, higher HbA1C and urinary albumin/creatinine ratio compared to those with normal stature *(p* < *0.05).* Additionally, patients with delayed puberty had higher HbA1c and dyslipidemia compared to those with normal puberty *(p* < *0.05).* The regression analysis revealed that factors associated with short stature were; age at diagnosis, HbA1C > 8.2, and albumin/creatinine ratio > 8 *(p* < *0.05).*

**Conclusion:**

Children with uncontrolled T1DM are at risk of short stature and delayed puberty. Diabetes duration and control seem to be independent risk factors for short stature.

## Introduction

Type 1 diabetes mellitus (T1DM) is a wasting disease characterized by severe deficiency or absence of insulin [1]. T1DM like other chronic disorders can interfere with growth and pubertal development [2]. T1DM usually begins in infancy, with a peak age of onset at 10 to 14 years. This can result in a longer duration of exposure to the consequences of hyperglycemia [3].

Dysregulation of the GH–IGF system is linked to T1DM. These alterations have been related to poor metabolic regulation and lack of endogenous insulin synthesis, resulting in decreased insulin carried in the portal vein, and subsequently inadequate insulin delivery to the liver. It has been reported that during the development of T1DM; IGF-1 and IGF-2 are lowered [4].

Significant bone development and remodeling is known to occur during childhood and adolescence; with adolescence accounting for 25% of peak bone mass [3]. Changes in bone microarchitecture were detected in adolescents with T1DM, which could explain the regularly reported loss in bone density and increased fracture risk in adults T1DM [5].

Puberty is the process of physical changes through which a child's body matures into an adult body capable of reproduction. On average, girls begin puberty at ages of 10–11 and complete puberty at ages 15–17, while boys generally begin puberty at ages 11–12 and complete puberty at ages 16–17 [6]. Puberty should be assessed annually in children and adolescents with T1DM. The pubertal stage is assessed by examination of the breasts and pubic hair in females, and genitals and pubic hair in males [7]. Severe growth retardation with pubertal delay, like in Mauriac syndrome, has been reported in patients with T1DM but this syndrome is now uncommon due to improvements in types of insulin therapy, as well as modes of insulin delivery and glycemic control [8].

We aimed to assess the anthropometric measures and pubertal growth in children and adolescents with T1DM regularly attending the Pediatric and Adolescents Diabetes Clinic (PADU), Ain Shams University. Our secondary aim was to detect risk determinants affecting these measures and their link to glycemic control.

## Subjects and methods

This cross-sectional observational study included 200 children and adolescents with T1DM attending the Pediatric and Adolescent Diabetes Unit (PADU), Pediatric Hospital. Patients with T1DM were defined according to the criteria of the International Society of Pediatric and Adolescent Diabetes *ISPAD 2022.* An informed consent in addition to an assent in adolescents was obtained from all patients and their legal guardians before enrollment in the study.

This study was reviewed and approved by the local research ethical committee in the Declaration of Helsinki (as revised in Brazil 2013). Approval of the ethical committee number is FWA 000017585 FMASU MS 151/2022.

Children and Adolescents with T1DM (aged 6 to 18 years old), with diabetes duration more than 2 years, were recruited. Children who have medical syndromes, and/or other chronic diseases, any clinical evidence of infection, hematological diseases, tumors, liver dysfunction, urinary tract disorders, connective tissue disease, or other autoimmune diseases or other types of DM (type 2 DM—MODY- diabetes secondary to a chronic disease) were excluded.

### Sampling

The sample size was calculated using PASS 11.0 and based on a study carried out by *Aljuhan et al.* [9]. In a one-way ANOVA study, sample sizes showed that fourteen patients (11.9%) were observed to have short stature, and 6.9% of children have wasting. The total sample of 163 subjects achieves 100% power to detect differences among the means versus the alternative of equal means using an F test with a 0.05000 significance level. However, the study will include 200 children to take into account 20% dropout rate.

### Methods

All recruited individuals were subjected to baseline clinical history, examination, and investigations. Baseline history included personal history (order of birth, family history of diabetes and socioeconomic state) and diabetes history (type of diabetes, diabetes duration and details of insulin therapy and frequency of hospital admission in the last 2 years). It also included history of diabetic complications in the form of acute and chronic complications, microvascular and macrovascular complications (atherosclerosis and coronary artery disease were assessed through history of palpitations, chest pain, and postural hypotension as well as blood pressure recordings from the medical files).

Detailed examination of all recruited individuals was done in the form of anthropometric measures and pubertal assessment. Anthropometric assessment included weight in kilograms (Kg), height in centimeters (cm), with calculation of weight and height SDS [10]. Body mass index (BMI) was calculated together with the calculation of BMI SDS according to the age and sex-specific reference values [11]***.***

Puberty was assessed by using Tanner staging [12]***.*** Pubertal assignments at each visit resulting from Tanner staging were based on breast maturation for girls and genital maturation for boys. Tanner stage 1 was considered prepubertal, and Tanner stages 2 through 5 were considered pubertal [11].

Fundus examination for assessment of diabetic retinopathy, in addition to simple rapid bedside neuropathy disability score (NDS) for diabetic peripheral neuropathy was carried out to identify any signs of microvascular complications. Complete examination including chest, heart and abdomen was also done for evidence of any diabetic complications.

### Investigations

- I- For all patients who were included in the study:
  - a- Complete blood count for detection of anemia.
  - b- Assessment of glycemic and metabolic control using HbA1c % which was assayed by immunoturbidimetric method done on Cobas 6000 autoanalyzer (Roche Diagnostics. 9115 Hague Road Indianapolis, IN46250) using reagents supplied by the company. The mean HbA1c% level of 2 readings in the last six months was calculated and used. Fasting serum triglycerides, total cholesterol, HDL and LDL were measured by absorbance photometry on Cobas Integra 800 (Roche Diagnostics GmbH, 68298 Mannheim, Germany) using reagents supplied by the company.
  - c- Thyroid and kidney function tests were done for all patients enrolled in the study.
  - d- Assessment of microvascular complications using an assay of microalbuminuria in urine sample was done on chemistry autoanalyzer Cobas c3ll (Roche Diagnostics GmbH, Indianapolis, IN46250, USA) by quantitative immunoturbidimetric assay using reagents supplied by the company. Microalbuminuria was defined as urinary albumin/creatinine ratio of 30–299 mg/g creatinine [13]. Fundus examination for retinopathy assessment was done by a professional single ophthalmologist through direct and indirect ophthalmoscopy.

- II- Patients with delayed growth or delayed puberty were further assessed:

Re-evaluation of autoimmune associations; thyroid profile (TSH & T3 & T4), celiac disease (Tissue Transglutaminase antibodies), and renal function tests (serum creatinine and serum electrolytes).

- 1- All patients with delayed growth were subjected to evaluation using
  - Serum Human insulin-like growth factors 1 (IGF-1) for detection of growth hormone deficiency using enzyme-linked immunosorbent assay (ELISA), Kit supplied by Bioassay laboratory technology. Levels were compared to age and sex-matched patients with diabetes having normal stature as a control group [14].
  - Plain x-ray on the left hand and wrist for radiological evaluation of bone age according to *Greulich and Pyle *[15]*.*

- 2- Patients with delayed puberty were subjected to baseline hormonal evaluation of sex hormones (testosterone and estradiol in boys and girls respectively) and gonadotropins (LH and FSH) followed by stimulation test (injection of S.C. GRH at 10 am then obtaining gonadotropins (LH and FSH) after 4 h of injection, and sex hormones (testosterone and estradiol in boys and girls respectively) after 24 h of injection [16].

### Statistical analysis

Data were collected, revised, coded, and entered into the Statistical Package for Social Science (IBM SPSS) version 23. The quantitative data were presented as mean, standard deviations and ranges when parametric and median, inter-quartile range (IQR) when data found non-parametric. Also, qualitative variables were presented as number and percentages.

The comparison between groups regarding qualitative data was done by using *Chi-square test* and/or *Fisher exact test* when the expected count in any cell found less than 5.

The comparison between two independent groups with quantitative data and parametric distribution was done by using an *Independent t-test* while non-parametric distribution was done by using *Mann–Whitney test.*

*Spearman correlation coefficients* were used to assess the correlation between two quantitative parameters in the same group.

The confidence interval was set to 95% and the margin of error accepted was set to 5%. So, the *p*-value was considered significant as the following: *P*-value > 0.05: Non-significant (NS), and *P*-value < 0.05: Significant (S).

## Results

Our study included 94 males and 106 females with a mean age of 13.18 ± 2.72 years, median (IQR) duration of diabetes of 5.13 (4 – 7.08) years, and mean insulin dose of 1.04 ± 0.27 U/Kg/day. The mean HbA1c was 9.76 ± 1.66%, with 30 patients (15.0%) having their levels below 7%.

The median (IQR) weight SDS of our patients was 0.29 (-0.31 – 0.76), height SDS was -0.25 (-0.71 – 0.31), BMI SDS was 0.62 (-0.01 – 1.23), waist circumference SDS was 0.67 (-0.1 – 1.19), hip circumference SDS was 0.56 (-0.24 – 1.12) and waist/hip ratio SDS was 0.52 (-0.23 – 1.31). Twenty two patients (11.0%) were classified as underweight (< -2 SDS), 174 (87.0%) had normal weight (-2 to 2 SDS) and 4 (2.0%) were classified as overweight (> 2 SDS).

In our study, we found that 12.5% of our patients (25 patients) were short and IGF-1 was low (IGF-1 SDS < -2) in 72% of them (18 patients). The Median (IQR) of IGF-1 SD was -2.7 (-3.6 – -1.2). We also found that patients with short stature have a significantly earlier age of onset of diabetes, earlier age at diagnosis and longer duration of diabetes compared to those with normal stature diabetic patients *(p* < *0.05)* as seen in Table 1. When comparing patients with short stature and those without short stature; we found that patients with short stature have significantly higher HbA1C and urinary albumin/creatinine ratio compared to those with normal stature patients. Meanwhile, patients with short stature had lower hemoglobin levels (Hb) and mean corpuscular volume (MCV) compared to those with normal stature *(p* < *0.05).* Additionally, patients with short stature had significantly lower IGF-1, IGF-1 SDS and delayed bone age compared to patients with normal stature as shown in Tables 2 and 3.

**Table 1:** Comparison between normal stature (no. = 175) and short stature (no. = 25) diabetic patients regarding sociodemographic data

		**Normal stature**	**Short stature**	**Test value**	***P*** **-value**
		**No. = 175**	**No. = 25**		
Sex	Male	84 (48.0%)	10 (40.0%)	0.562^a^	0.453
	Female	91 (52.0%)	15 (60.0%)		
Age (years)	Mean ± SD	13.21 ± 2.68	12.95 ± 3.06	0.447^b^	0.655
	Range	6.25 – 17.75	7 – 17.67		
Paternal consanguinity	No	137 (78.3%)	18 (72.0%)	1.037^a^	0.595
	Yes	38 (21.7%)	7(28.0%)		
Similar conditions	No	132 (75.4%)	15 (60.0%)	2.673^a^	0.102
	Yes	43 (24.6%)	10 (40.0%)		
socioeconomic status	Low	74 (42.5%)	14 (56.0%)	2.811^a^	0.245
	Middle	88 (50.6%)	11 (44.0%)		
	High	12 (6.9%)	0 (0.0%)		
Age at diagnosis (years)	Median (IQR)	7 (5.42 – 10.17)	4.33 (3.17 – 6.08)	-4.845^c^	0.000
	Range	2 – 14	2 – 8.42		
Duration of T1DM (years)	Median (IQR)	5 (4 – 7)	8 (6.42 – 10)	-5.159^c^	0.000
	Range	2.25 – 13	5 – 14		

*P*-value > 0.05: Non-significant

*P*-value < 0.05: Significant

*P*-value < 0.01: Highly significant

^a^Chi-square test

^b^Independent t-test

^c^Mann-Whitney test

**Table 2:** Comparison between normal stature (no. = 175) and short stature (no. = 25) diabetic patients regarding biochemical data

			**Normal stature**	**Short stature**	**Test value**	***P*** **-value**
			**No. = 175**	**No. = 25**		
**CBC**	Hb (g/dl)	Mean ± SD	12.16 ± 0.96	11.20 ± 0.83	4.753^a^	0.000
		Range	9.8 – 14.1	9.8 – 13.1		
	MCV(fl)	Mean ± SD	81.97 ± 3.41	79.88 ± 2.82	2.922^a^	0.004
		Range	71 – 91	73 – 85		
	PLT (10^9^/L)	Mean ± SD	267.61 ± 50.61	266.80 ± 39.34	0.076^a^	0.939
		Range	151 – 515	205 – 317		
	WBC(10^9^/L)	Mean ± SD	5.99 ± 1.08	6.26 ± 0.98	-1.161^a^	0.247
		Range	3.2 – 9.7	4.9 – 8.3		
HbA1C%		Mean ± SD	7.92 ± 1.00	9.76 ± 1.66	-7.782^a^	0.000
		Range	5.9 – 12.8	7.6 – 14		
CRP (mg/dl)		Median (IQR)	5.4 (2.8 – 6.4)	4.8 (1.65 – 6)	-1.604^b^	0.109
		Range	0.6 – 26	0.3 – 12		
ESR (mm/hr)		Median (IQR)	12 (10 – 14)	13 (10 – 15)	-1.134^b^	0.257
		Range	8 – 22	8 – 22		
Creatinine (mg/dl)		Mean ± SD	0.78 ± 0.11	0.74 ± 0.19	1.684^a^	0.094
		Range	0.1 – 1.1	0.45 – 1.1		
ALT (IU/L)		Median (IQR)	11 (9 – 13)	11 (9 – 12)	-0.274^b^	0.784
		Range	6 – 77	9 – 22		
AST (IU/L)		Median (IQR)	17 (16 – 19)	17 (15 – 18)	-0.910^b^	0.363
		Range	11 – 70	12 – 23		
**Lipid profile**	Fasting cholesterol (mg/dl)	Mean ± SD	146.02 ± 49.82	167.00 ± 68.00	-1.874^a^	0.062
		Range	85 – 318	76 – 289		
	HDL (mg/dl)	Mean ± SD	33.32 ± 15.51	36.72 ± 19.00	-0.995^a^	0.321
		Range	9 – 98	18 – 80		
	LDL (mg/dl)	Mean ± SD	68.07 ± 38.85	76.48 ± 32.50	-1.032^a^	0.303
		Range	29 – 188	39 – 156		
	TG (mg/dl)	Mean ± SD	91.15 ± 45.64	108.12 ± 49.19	-1.722^a^	0.087
		Range	33 – 312	48 – 199		
Urinary albumin/creatinine ratio		Median (IQR)	2.9 (1.6 – 8.5)	33 (9 – 64)	-5.550^b^	0.000
		Range	0.3 – 34	0.6 – 133		

*HDL* high density lipoprotein, *LDL* Low density lipid lipoprotein, *TG* triglycerides, *TTG* tissue transglutaminase, *FT4* free thyroxine. *TSH* thyroid stimulating hormone, *ESR* erythrocytes sedimentation rate, *CRP* C-reactive protein

*P*-value > 0.05: Non-significant

*P*-value < 0.05: Significant

*P*-value < 0.01: Highly significant

^a^Independent t-test

^b^Mann-Whitney test

**Table 3:** Comparison between normal stature diabetic patients (no. = 175) and short stature (no. = 25) regarding IGF-1, IGF-1% according to age and sex, IGF-1 SD, IGF-1% according to Tanner stage and bone age (years)

		**Normal stature**	**Short stature**	**Test value**	***P*** **-value**
		**No. = 175**	**No. = 25**		
IGF-1	Median (IQR)	375 (315 – 421)	87 (55 – 201)	-2.700^c^	0.007
	Range	305 – 451	25 – 608		
IGF-1	abnormal IGF-1	0 (0.0%)	18 (72.0%)	9.000^a^	0.003
	normal IGF-1	20 (100.0%)	7 (28.0%)		
IGF-1% according to age and sex	(0.1—2.5)	0 (0.0%)	18 (72.0%)	10.560^a^	0.014
	(2.5—50)	8(40.0%)	3 (12.0%)		
	(50—95)	12 (60.0%)	3 (12.0%)		
	(95—97.5)	0 (0.0%)	1 (4.0%)		
IGF-1 SD	Median (IQR)	0.1 (-0.9 – 0.3)	-2.7 (-3.6 – -1.2)	-2.283^c^	0.022
	Range	-1.1 – 0.3	-4.4 – 1.6		
IGF-1% according to Tanner stage	(0.1—2.5)	0 (0.0%)	16 (64.0%)	9.600^a^	0.008
	(2.5—50)	4(20.0%)	5 (20.0%)		
	(50—97.5)	16 (80.0%)	4 (16.0%)		
Bone age (years)	Mean ± SD	15.60 ± 1.95	11.48 ± 3.24	2.721^b^	0.011
	Range	13 – 17	6 – 17		

*IGF-1* insulin growth factor 1

*P*-value > 0.05: Non-significant

*P*-value < 0.05: Significant

*P*-value < 0.01: Highly significant

^a^Chi-square test

^b^Independent t-test

^c^Mann-Whitney test

Fifteen patients were found to have delayed puberty. Their median (IQR) stimulated LH was 38.5 IU/L (9.5 – 43.9), stimulated FSH was 17.3 IU/L (7.6 – 19.8), stimulated estradiol was 219.9 pg/ml (31.9 – 281.6) and the mean (SD) stimulated total testosterone was 328.55 ± 23.97 ng/dL. There was a significant increase in the incidence of delayed puberty in females compared to males (86.7% versus 13.3% respectively). When comparing patients with delayed puberty to those with normal puberty, we found that patients with delayed puberty had significantly lower weight SDS [median (IQR) = -2.04 (-2.98 – 0.36) versus 0.29 (-0.18 – 0.81) respectively], height SDS [median (IQR) = -2.9 (-3.85 – -0.53) versus -0.09 (-0.61 – 0.37) respectively], BMI SDS [median (IQR) = 0.28 (-0.86 – 1.1) versus 0.62 (0.08 – 1.25) respectively] and waist/hip ratio SDS [median (IQR) = 1.93 (0.26 – 2.23) versus 0.48 (-0.27 – 1.27) respectively] *(p* < *0.05).* They also had significantly higher HbA1C, fasting cholesterol, HDL, LDL, TG and urinary albumin/creatinine ratio compared to those with normal puberty. Meanwhile, patients with delayed puberty had significantly lower Hb and MCV compared to those with normal puberty as shown in Table 4.

**Table 4:** Comparison between normal puberty (no. = 185) and delayed puberty diabetic patients (no. = 15) regarding biochemical data

			**Normal puberty**	**Delayed puberty**	**Test value**	***P*** **-value**
			**No. = 185**	**No. = 15**		
**CBC**	Hb(g/dl)	Mean ± SD	12.12 ± 0.96	11.00 ± 0.76	4.401^a^	0.000
		Range	9.8 – 14.1	9.8 – 13.1		
	MCV (fl)	Mean ± SD	81.86 ± 3.43	79.87 ± 2.61	2.197^a^	0.029
		Range	71 – 91	76 – 85		
	PLT (10^9^/L)	Mean ± SD	267.16 ± 49.70	271.80 ± 44.67	-0.350^a^	0.726
		Range	151 – 515	215 – 345		
	WBC (10^9^/L)	Mean ± SD	5.99 ± 1.06	6.43 ± 1.07	-1.524^a^	0.129
		Range	3.2 – 9.7	4.9 – 8.2		
HbA1C%		Mean ± SD	8.04 ± 1.23	9.50 ± 0.66	-4.530^a^	0.000
		Range	5.9 – 14	8.6 – 10.5		
CRP (mg/dl)		Median (IQR)	6 (2.8 – 6.4)	1.85 (1.5 – 6)	-2.628^b^	0.009
		Range	1.3 – 26	0.3 – 12		
ESR (mm/hr)		Median (IQR)	12 (10 – 14)	12 (9 – 15)	-0.243^b^	0.808
		Range	8 – 22	8 – 22		
Creatinine (mg/dl)		Mean ± SD	0.77 ± 0.11	0.83 ± 0.16	-1.910^a^	0.058
		Range	0.1 – 1.1	0.45 – 1.1		
ALT (IU/L)		Median (IQR)	11 (9 – 13)	12 (10 – 14)	-1.128^b^	0.259
		Range	6 – 77	9 – 22		
AST (IU/L)		Median (IQR)	17 (16 – 19)	17 (15 – 19)	-0.440^b^	0.660
		Range	11 – 70	12 – 23		
**Lipid profile**	Fasting cholesterol(mg/dl)	Mean ± SD	146.48 ± 49.79	175.33 ± 77.82	-2.056^a^	0.041
		Range	85 – 318	76 – 287		
	HDL (mg/dl)	Mean ± SD	32.90 ± 14.98	44.13 ± 23.53	-2.658^a^	0.009
		Range	9 – 98	19 – 80		
	LDL (mg/dl)	Mean ± SD	66.91 ± 37.34	96.40 ± 38.60	-2.935^a^	0.004
		Range	29 – 188	57 – 175		
	TG (mg/dl)	Mean ± SD	91.12 ± 45.27	119.80 ± 52.29	-2.332^a^	0.021
		Range	33 – 312	54 – 199		
Urinary albumin/creatinine ratio		Median (IQR)	3 (1.6 – 10)	28 (11 – 49)	-3.791^b^	0.000
		Range	0.3 – 133	0.6 – 70		

*HDL* high density lipoprotein, *LDL* Low density lipid lipoprotein, *TG* triglycerides, *TTG* tissue transglutaminase, *FT4* free thyroxine. *TSH* thyroid stimulating hormone, *ESR* erythrocytes sedimentation rate, *CRP* C-reactive protein

*P*-value > 0.05: Non significant

*P*-value < 0.05: Significant

*P*-value < 0.01: Highly significant

^a^Independent t-test

^b^Mann-Whitney test

IGF-1 was positively correlated with the duration of diabetes (years.), weight (kg), height (cm), height SDS, waist circumference, waist circumference SDS, hip circumference, waist/hip waist/hip SDS, penile length (cm), right testicular volume (ml), left testicular volume (ml), and Tanner score as shown in Table 5.

**Table 5:** Correlation between IGF-1 and each of duration of diabetes (years), weight (kg), height (cm), height SDS, BMI (kg/m2), BMI SDS, waist circumference, Waist circumference SDS, Hip circumference, Hip circumference SDS, waist /hip ratio, waist /hip SDS, penile length (cm), right testicular volume (ml), left testicular volume (ml) and Tanner score

	**IGF-1**	
	**r**	***P*** **-value**
Duration of Diabetes (years)	**0.452**	**0.023**
Weight (kg)	**0.377**	**0.040**
Height (cm)	**0.580**	**0.001**
Height SDS	**0.465**	**0.019**
Waist circumference (cm)	**0.525**	**0.003**
Waist circumference SDS	**0.624**	**0.000**
Hip circumference (cm)	**0.504**	**0.004**
waist/hip SDS	**0.394**	**0.031**
Penile length (cm)	**0.859**	**0.001**
Right testicular volume (ml)	**0.732**	**0.016**
Light testicular volume (ml)	**0.732**	**0.016**
Tanner score	**0.570**	**0.003**

Spearman correlation coefficient

*P*-value > 0.05: Non significant

*P*-value < 0.05: Significant

*P*-value < 0.01: Highly significant

The univariate and multivariate logistic regression analysis showed that factors significantly associated with short stature were; age at diagnosis *(p-value* = *0.007)*, HbA1C > 8.2 *(p-value* = *0.001)*, and albumin/creatinine ratio > 8 *(p-value* = *0.003).*

**Fig. 1:**
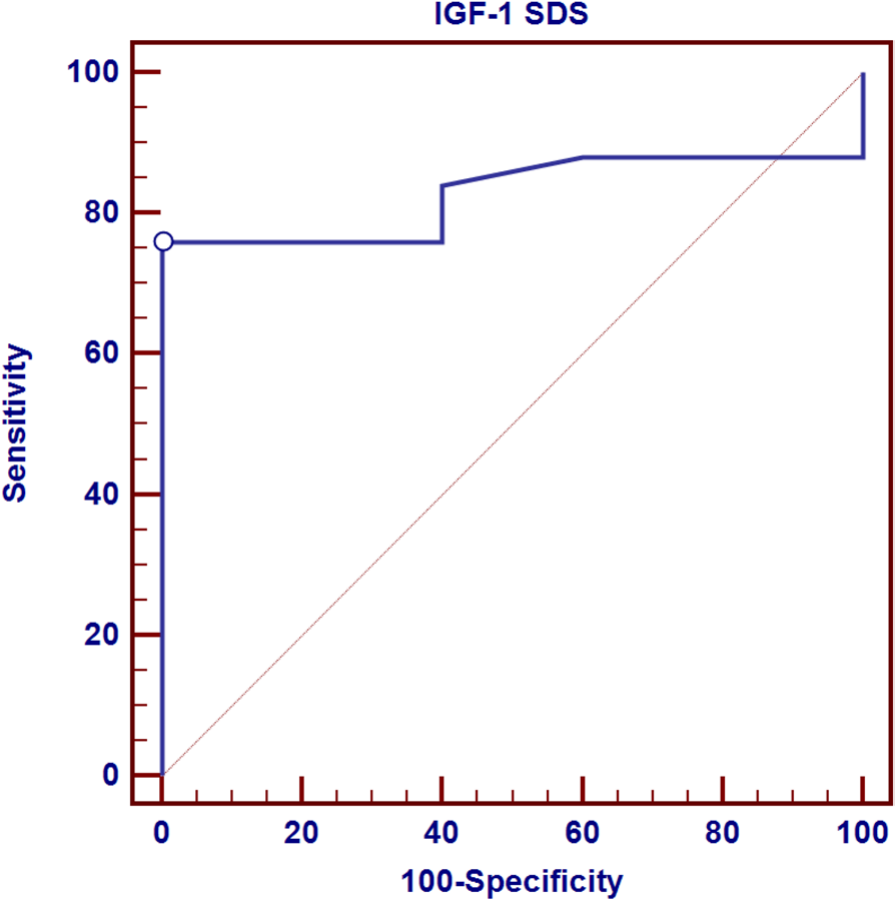
ROC curve for IGF-1 SDS to detect short stature

Receiver operating characteristic curve (ROC) showed that the best cut-off point of IGF-1 SDS to detect short stature was found to be ≤ -1.2 with a sensitivity of 76%, specificity of 100.0%, PPV of 100.0%, NPV of 45.5% and total accuracy of 82.8% as seen in Fig. 1.

## Discussion

In our study, we found that the prevalence of short stature among our studied cases was (12.5%) and their mean serial HbA1c% was 9.76 ± 1.66. Comparable to our results, a previous study done in Egypt by *El-Ella *et al*.* revealed that the prevalence of short stature was 16.3% among their patients and their mean serial HbA1c% was 10.53 ± 2.1. The higher HbA1c among their patients could explain the slightly higher prevalence of short stature in their center [17]. Our results were also comparable to those obtained by *Aljuhani *et al*.* in Saudi Arabia who found that the percentage of short stature among studied participants was (11.9%) and their mean serial HbA1c% was 9.15 [9]. Additionally, *Sap *et al*.* found that in Cameroonian children with T1DM, the percentage of short stature was (10%) and their mean serial HbA1c% was 11.5 [18]. Meanwhile, *Balde *et al*.* found a higher prevalence of short stature among children and adolescents in Guinea (28%) and their mean serial HbA1c% was 10.3. This could be explained by chronic nutritional insufficiency and starvations in Guinea, which could additionally have a more negative impact on growth and stature [19]. On the other hand, studies done in in Europe as evaluated by *Bonfig and his colleagues* found that the percentage of short stature among patients recruited from specialized centers in Germany and Austria was (9.2%) and their mean serial HbA1c% was 7.9 ± 1.2. The lower percentage of short stature in developed countries could be explained by better nutrition and better glycemic control as evidenced by the lower HbA1c [20].

Short stature in diabetic patients could be related to metabolic control (HbA1c). In our study, we found that short stature in diabetic patients was significantly related to higher HbA1C compared to those with normal stature. In agreement with *Zurita-Cruz *et al*.,* who reported in their study that HbA1c and poor glycemic control were lower in patients with normal growth parameters compared to those with growth alterations [21]. On the other hand, several studies found that there was no significant correlation between metabolic control and linear growth in diabetic boys or girls. This could indicate that the diabetic control, as reflected by HbA1c levels might not be the only determinant of short stature in children with TIDM [9, 22–24].

Interestingly, we illustrated that short stature in our patients was significantly related to earlier age at diagnosis and longer duration of diabetes compared to those with normal stature. It was previously shown that adult height was negatively correlated to the duration of diabetes [20, 25–27]. On the other side, some studies concluded that the decrease in height gain was independent of the duration of TIDM or the degree of metabolic control. With the improvement in therapeutic regimen, regular diet and physical activity; normal growth should be expected in children with TIDM [22, 23]. However, data on growth and pubertal development still remain controversial, and above all, it is unclear whether the degree of metabolic control or the duration of the disease affects final height.

Short stature in diabetic patients could be also related to anthropometrical measures. In our study, we found that short stature was related to lower weight, weight SDS, height SDS, BMI SDS, waist circumference, hip circumference, and Hip circumference SDS compared to diabetic patients with normal stature. Similarly, several previous studies revealed that anthropometrical measurements (weight, height, and BMI) were significantly low among diabetic patients [19, 28–30]. On the contrary, *Clarson* et al***.*** studied the growth and pubertal development in 122 children, and found no correlation between diabetes and the mean height or weight percentiles. Proper nutrition is needed to achieve normal anthropometrical measurements and adequate height velocity according to age group for diabetic patients, which could be lacking in our developing countries [22]. Furthermore, deficiency of regular celiac screening in developing countries could be a cause of underbuilt, poor weight gain and, subsequently short stature. However, none of our studied patients had positive antibodies for celiac disease at the time of recruitment.

Short stature in diabetic patients could be also related to lower IGF-1 levels. In our study, we cleared that diabetic patients with short stature had significantly lower IGF-1 compared to those with normal stature (72% versus 28% respectively). Similarly, numerous studies have reported that poor height velocity in children with T1DM seems to be linked to their reduced IGF-1 serum level compared to healthy peers [31–34]. Adequate insulin secretion is needed to maintain normal serum concentrations of IGF-1 and IGFBPs and indirectly promote growth [35].

Furthermore, the Receiver operating characteristic curve (ROC) showed that the best cut-off point of IGF-1 SD to detect short stature was found to be ≤ -1.2 with a sensitivity of 76%, specificity of 100.0%, PPV of 100.0%, NPV of 45.5% and total accuracy of 82.8%. *Felício* et al.also found that levels of IGF-1 alone presented high accuracy for the diagnosis of short stature, with the cut-off point − 2SD showing the best results due to higher sensitivity (84.6%) [36]. Our results are in accordance with a meta-analysis performed by *Shen* et al***.*** with 12 studies, who reported a specificity of 69% for IGF-1 in the diagnosis of short stature when using the − 2SD as cut-off [37]. *Cianfarani* et al***.*** evaluated 33 patients with growth hormone deficiency (GHD) and 56 subjects with idiopathic short stature (non-GHD) and found that the IGF-1 SDS cut-off of − 1.9 best discriminated GHD from idiopathic short stature, with 73% sensitivity and 95% specificity [38]. GH stimulation tests do not demonstrate perfect sensitivity or specificity. This has motivated researchers to look for more promising diagnostic tools. Single randomly obtained serum measurements of IGF-1 could reflect the circulating GH, attracting the endocrine community as an alternative to GH stimulation tests [39].

The IGF-1 levels could also be related to pubertal status, where in our study we illustrated that diabetic patients with delayed puberty and short stature had significantly lower IGF-1 levels. Similarly, *Zhao* et al***.*** found that abnormal IGF-1 was significantly associated with delayed puberty and short stature *(p* < *0.01)* [40]. However; in our patients, IGF-1 levels compared according to Tanner stage showed that there was no significant difference, indicating the relevant impact of IGF-1 regardless of the Tanner stage.

In this study, we found that there was a significant positive correlation between IGF-1 levels and each of the duration of diabetes (years) and anthropometrical measures. Similarly, *Raman* et al***.*** found that serum IGF-1 levels had a significantly positive correlation with the duration of diabetes *(p* < *0.05)* [41]. Other studies found a significant positive association between the rate of increase in weight and height during childhood and serum IGF-1 levels [42, 43].

Regarding univariate and multivariate logistic regression analysis for the factors associated with short-stature diabetic patients, we found that the most significantly associated factors with short stature were; age at diagnosis, HbA1c > 8.2, and albumin/creatinine ratio > 8. Similarly, regression analysis of a previous study revealed that there was a significant positive correlation of height to the age at diagnosis [44]. Also, *Hassan and colleagues* found that diabetic-controlled children were taller and heavier, with higher BMI and lower albumin/creatinine ratio than those of the diabetic uncontrolled children in Egypt [45].

In our study, we found that patients with short stature have significantly delayed puberty and delayed bone age compared to those with normal stature patients. These results are in agreement with an earlier study which reported that full sexual maturation in T1D boys (stage 5) is retarded and occurs at a mean age of 17.2 years [46]. Also, *Attia and colleagues* found that T1D causes marked retardation of puberty, where nearly 25% of the diabetic boys were not sexually mature [28]. Meanwhile, *Zurita-Cruz *et al*.* reported that 50% of their patients were prepubertal, and only 5% had reached a Tanner stage 4 [21]. Similarly, another study carried on 206 adolescents with T1DM, showed significant impairment in pubertal growth with height SDS reduction [47]. On the other hand, *Zhao *et al*.* found that diabetic patients with delayed puberty showed significantly higher bone age than diabetic patients without delayed puberty. They concluded that bone age is not always related to cases with delayed puberty [48].

In our study, we found that the prevalence of delayed puberty was 7.5%. Patients with delayed puberty had a significantly longer duration of diabetes compared to those with normal puberty. Similarly, *Elamin *et al*.* found that the retardation in physical growth and pubertal development in males and females was positively correlated with the duration of diabetes before the onset of puberty and HbA1c concentration [25]. Interestingly, female patients in our study had significant delayed puberty compared to males (86.7% versus 13.3% respectively). Similar results were previously obtained by *Oza *et al*.* who studied a group of Indian children and adolescents [49]. Ovarian function appears to be modified in adolescents with T1DM [50].

We illustrated that diabetic patients with delayed puberty had significantly higher BMI SDS and waist/hip SDS compared to those patients with normal puberty. Similarly, *Boulbaroud *et al*.* showed that pubertal delay increases with HbA1c and BMI [51]. On the other hand, *Aljawarneh *et al. found that girls with T1DM are more likely to be obese or overweight and the menarche age decreases as the percentile of BMI increases and vice versa [34]. Meanwhile, other earlier studies revealed that BMI was not associated with delayed puberty [52, 53]. Furthermore, we found that diabetic patients with delayed puberty had significantly higher HbA1c, fasting cholesterol, HDL, LDL and TG compared to those diabetic patients with normal puberty. Similarly, *Plamper *et al*.* found that diabetic patients with delayed puberty had significantly higher HbA1C, fasting cholesterol, HDL, LDL, TG compared to those with normal puberty [2]. Thus, the high BMI in our patients with delayed puberty could be related to other factors such as type of diet, inadequate exercise and daily habits, especially in female adolescents, rather than the pubertal stage.

In our study, we found that diabetic patients with delayed puberty had a higher albumin/creatinine ratio compared to those with normal puberty. Furthermore, similar to our study, ***Zhao ***et al***.*** found that there was a significant negative correlation between estradiol and albumin/creatinine ratio. We don’t know if this could explain the higher incidence of delayed puberty among females in our study [40].

## Conclusion

Short stature and delayed puberty are common complications detected in uncontrolled children and adolescents with T1DM. Thus, healthcare planners and providers should be aware of the importance of regular growth and pubertal assessment to individuals with T1DM.

## Data Availability

All data generated or analyzed during this study are included in this published article. All data are available for sharing.
